# Cytokine profile and cytoskeletal changes after herpes simplex virus type 1 infection in human trabecular meshwork cells

**DOI:** 10.1111/jcmm.16862

**Published:** 2021-09-01

**Authors:** Jin A. Choi, Hyun‐hee Ju, Ju‐Eun Kim, Jiyoung Lee, Donghyun Jee, Chan Kee Park, Soon‐young Paik

**Affiliations:** ^1^ Department of Ophthalmology College of Medicine St. Vincent’s Hospital The Catholic University of Korea Seoul Korea; ^2^ Department of Microbiology College of Medicine The Catholic University of Korea Seoul Korea; ^3^ Department of Ophthalmology College of Medicine Seoul St. Mary’s Hospital The Catholic University of Korea Seoul Korea

**Keywords:** glaucoma, herpes virus, HSV_1, intraocular pressure, MCP‐1, uveitis

## Abstract

Uveitis caused by herpes simplex virus (HSV)‐1 is characterized by increased intraocular pressure (IOP) in the presence of anterior chamber inflammation. Despite their clinical significance, the pathogenic changes associated with HSV‐1 infection in trabecular meshwork (TM) cells, the key cell type regulating IOP, have not been completely elucidated. In this study, cytokine array analyses showed a significant stepwise increase in monocyte chemoattractant protein (MCP)‐1 expression upon HSV‐1 infection in TM cells (*p* < 0.05). HSV‐1 infection led to downregulation of fibrogenic molecules (fibronectin, α‐smooth muscle actin, connective tissue growth factor and TGF‐β1). Notably, HSV‐1 infection caused a significant increase in actin stress fibres, with a twofold increase in active RhoA, which was enhanced by treatment with TGF‐β1 and inhibited by treatment with the Rho‐kinase inhibitor, Y‐27632. TM cells treated with MCP‐1 exhibited a dose‐dependent increase in actin stress fibres compared to untreated TM cells. Our study suggests that HSV‐1 infection in TM cells increases cell contractile activity rather than fibrotic changes in the extracellular matrix (ECM) components. Taken together, these observations demonstrate the enhanced expression of MCP‐1 and TM cell contractile activity upon HSV‐1 infection and events with potential implications for the pathobiology of abrupt IOP elevation in HSV‐1 anterior uveitis.

## 1 INTRODUCTION

Anterior uveitis is the most common type of intraocular inflammation and has both infectious and non‐infectious aetiologies.^1^The herpes viruses are among the most frequent causes of viral uveitis worldwide.^2^ Members of the Herpesviridae family, which includes herpes simplex virus (HSV)‐1, varicella zoster virus (VZV) and cytomegalovirus (CMV), have lifelong latency after primary infection. HSV‐1 is a particularly prevalent ocular pathogen infecting 60–90% of the world's population.^3^ The clinical spectrum of recurrent ocular disease induced by HSV‐1 predominantly involves the anterior segment of the eye.^4^

Patients with anterior uveitis caused by HSV‐1 infection are particularly prone to the development of glaucoma,^5^ characterized by markedly increased IOP in the presence of anterior chamber inflammation which is normalized after corticosteroid treatment or cessation of the inflammation. In specimens of patients with herpes simplex kerato‐iritis, a thickened trabecular band infiltrated with fibrin and inflammatory cells has been histologically documented.^6^ A herpetic kerato‐uveitis rabbit model also showed acute elevation of IOP accompanied by severe infiltration of inflammatory cells in the iris root and trabecular meshwork (TM).^7^ These findings suggest that direct inflammation of TM cells plays a pathogenic role in the acute elevation of IOP in patients with HSV‐1 anterior uveitis. Human TM cells, the key cells involved in the regulation of IOP, effectively support HSV‐1 replication.^8^

TM cells exhibit two major functions, that is, filtration and resistance generation. Resistance by the trabecular outflow pathway depends on the amount of actin cytoskeleton contraction in the TM cells and accumulation of extracellular matrix (ECM) material in the pathway.^9^ TM cells, which share features of fibroblasts, secrete ECM molecules and degradative enzymes for the continuous remodelling of ECM material. Although ECM remodelling is actively processed in the outflow pathway, the turnover time of matrix protein is about 48 h.^9^, ^10^ It has been shown that RhoA signalling mediates the early IOP rise induced by increased contractile activity in the TM cells but not the sustained IOP elevation caused by increased fibrosis in the outflow pathway.^11^ Therefore, considering the abrupt changes in IOP associated with inflammation in HSV‐1 uveitis, it is possible that HSV‐1 predominantly induces cytoskeletal changes in TM cells rather than the accumulation of ECM in the outflow pathway. However, the host cell response and cytoskeletal changes upon HSV‐1 infection in TM cells have not been elucidated.

In this study, we investigated the cytokine profile and cytoskeletal changes upon HSV‐1 infection in human TM cells. Using the molecules identified in the analyses, we further investigated potential target cytokines in HSV‐1 anterior uveitis.

## 2 MATERIALS AND METHODS

### 2.1 Materials

The following materials were used: recombinant human transforming growth factor (TGF)‐β1, monocyte chemoattractant protein (MCP)‐1 and MCP‐1 ELISA kits with pre‐coated plates from R&D Systems; dexamethasone (DEX) from Sigma‐Aldrich; the Rho‐associated kinase (ROCK) inhibitor Y‐27632 from EMD Millipore; the murine monoclonal anti‐RhoA antibody ab54835 from Abcam; rhodamine phalloidin from Invitrogen; the Rho Activation Assay Biochem Kit from Cytoskeleton; goat anti‐mouse antibody (VectaFluor R.T.U. DyLight 488 anti‐mouse); and 4′,6‐diamidino‐2‐phenylindole (DAPI) (VECTASHIELD^®^ with DAPI) from Vector Laboratories.

### 2.2 Cells and viruses

The National Culture Collection for Pathogens (NCCP) HSV‐1 clinical strain no. 43002 was generously provided by the Korea Centers for Disease Control and Prevention (Osong, Republic of Korea; http://www.cdc.go.kr/CDC/eng/main.jsp). Vero cells were cultured in Dulbecco's modified Eagle's medium (DMEM) with 10% foetal bovine serum (FBS). DMEM supplemented with 1% FBS was used for infection and other treatments of cells. HSV‐1 NCCP no. 43002 was propagated in Vero cells,^12^ and standard plaque titrations were performed on Vero cells.^8^ The percentage identities of major genes between HSV‐1 NCCP no. 43002 and HSV‐1 KOS strain are shown in Supplementary information 1. We obtained primary human TM cells from ScienCell Research Labs. TM cells were cultured at 37℃ under 5% CO_2,_ in TM cell medium (ScienCell Research Labs).^13^ Characterization of TM cells are shown in Supplementary information 2. TM cells from the fourth or fifth passage were seeded in 6‐well plates and allowed to grow to confluence.

To infect primary TM cells with HSV‐1, the TM cells were adsorbed with the virus stock for 1 h at a multiplicity of infection (MOI) of 1 or 5 and re‐fed with fresh DMEM supplemented with 1% FBS. Analyses of HSV‐infected TM cells were performed 12 h or 2 days post‐infection (PI). In some experiments, infected and mock‐infected TM cell cultures were exposed to recombinant TGF‐β1 (15 ng/ml), Y‐27632 (10 μM), DEX (100 nM) and MCP‐1 (10 or 50 ng/ml) for 12 h. The study protocols were approved by the Institutional Review Board at the Catholic University of Korea in accordance with the Declaration of Helsinki for experiments involving human tissues and samples (local IRB No.VC18ZNSI0062).

### 2.3 Cytokine array

To obtain a global view of cytokine expression associated with HSV‐1 infection, supernatants of mock‐infected and HSV‐1‐infected TM cells (at a MOI of 1 or 5) were collected 12 h PI and assayed using a protein array system (Human Cytokine Antibody Array C5; RayBiotech), which determined the levels of expression of 80 cytokines related to inflammation. The intensities of the chemiluminescence signals were converted into numbers using LAS‐1000 plus with MultiGauge software (Fujifilm) and normalized relative to positive control signals.

### 2.4 Rho activation assay

The TM cells were cultured on 10 cm diameter dishes (surface area 56.7 cm^2^). After reaching confluence, cells were infected with HSV‐1 at a MOI of 1. At 12 h PI, RhoA activity was assessed by a pull‐down assay with a Rho Activation Assay Biochem Kit (BK036; Cytoskeleton), in accordance with the manufacturer's instructions. Briefly, Rhotekin‐Rho binding domain beads were used as an indicator of active RhoA. The beads were washed three times with lysis buffer, and the bound GTP‐Rho was detected by immunoblotting analyses with mouse anti‐human monoclonal IgM RhoA antibody (ARH04; Cytoskeleton; 1:500), followed by goat anti‐mouse horseradish peroxidase‐conjugated secondary antibody (1:2000). Active RhoA (GTP binding form)‐immunoreactive bands were visualized using ECL Advanced Western Blotting Detection Reagent (GE Healthcare) and determined using a luminescent image analyser (LAS‐4000 mini; Fujifilm). Densitometry of immunoreactive bands was performed using ImageJ software (National Institutes of Health).

### 2.5 Viral DNA replication assays

HSV‐1 viral DNA was harvested from infected or mock‐infected TM cells at 12 h or 2 days PI in the presence or absence of various treatments. Total DNA was isolated using a commercially available kit (QIAmp DNA Mini Kit; Qiagen, Hilden, Germany).^14^, ^15^ Briefly, cells grown in a monolayer were detached from the culture plate by trypsinization. The cells were centrifuged for 5 min at 300 × *g*, and the cell pellet was resuspended in phosphate buffered saline (PBS) to a final volume of 200 µl. Then, the samples were treated according to the protocols for ‘DNA purification from blood or body fluids’ suggested by the manufacturer. The viral DNA from each sample was quantified by real‐time quantitative PCR using nucleotide primers proven specific for the HSV‐1 DNA polymerase gene (forward: 5′‐CATCACCGACCCGGAGAGGGAC‐3′ and reverse: 5′‐GGGCCAGGCGCTTGTTGGTGTA‐3′).^16^, ^17^ Real‐time PCR with primers for β‐actin was also performed to serve as an internal control for input DNA.

### 2.6 Real‐time PCR and Enzyme‐linked immunosorbent assay (ELISA)

From the mock‐infected or HSV‐1 infected TM cells with or without various treatments, total RNA was extracted using an RNeasy Mini Kit (Qiagen). The total RNA was quantified and reverse‐transcribed to cDNA using PrimeScript RT reagent Kit (Takara Bio). The relative expression levels of mRNAs were determined using a Roche Diagnostics LightCycler 2.0 Real‐Time PCR System (Roche). The sequences of the real‐time PCR primer pairs are shown in Table 1. To ensure equal loading and amplification, all products were normalized relative to β‐actin transcript as an internal control.

**TABLE 1:** Sequences of forward and reverse primer sets used for real‐time PCR

Amplification	Forward primer	Reverse primer
α‐SMA	5′ ‐GACAATGGCTCTGGGCTCTGTAA−3′	5′ ‐CTGTGCTTCGTCACCCACGTA−3′
Collagen1A	5′ ‐GGAATGAAGGGACACAGAGG−3′	5′ ‐TAGCACCATCATTTCCACGA−3′
CTGF	5′ ‐CTCCTGCAGGCTAGAGAAGC−3′	5′ ‐GATGCACTTTTTGCCCTTCTT−3′
Fibronectin	5′ ‐CTGGCCGAAAATACATTGTAA−3′	5′ ‐CCACAGTCGGGTCAGGAG−3′
TGF‐β1	5′ ‐GAGCCTGAGGCCGACTACTA−3′	5′‐GGGTTCAGGTACCGCTTCTC−3′
TGF‐β2	5′ ‐GTCGCGCTCAGCCTGTCT−3′	5′‐CCTCGATCCTCTTGCGCAT−3′
MCP−1	5′ ‐CTGAAGCTCGTACTCTC−3′	5′‐CTTGGGTTGTGGAGTGAG−3′
FAK	5′‐GTGCTCTTGGTTCAAGCTGGA−3′	5’‐ACTTGAGTGAAGTCAGCAAGATGTG−3′
Paxillin	5′‐ACGTCTACAGCTTCCCCAACAA−3′	5′‐AGCAGGCGGTCGAGTTCA−3′
β‐actin	5′ ‐GTCCACCTTCCAGCAGATGT−3′	5′ ‐AAAGCCATGCCAATCTCATC−3′

Abbreviations: CTGF: connective tissue growth factor; FAK: focal adhesion kinase; MCP: monocyte chemoattractant protein; TGF: transforming growth factor; α‐SMA: smooth muscle actin.

Levels of secreted monocyte chemotactic protein (MCP)‐1 were measured using a commercially available MCP‐1 ELISA Kit with pre‐coated plates (R&D Systems). Conditioned medium was harvested, cleared by centrifugation and stored at –70℃. The medium was subsequently acid‐activated and directly assayed using an ELISA plate reader at 450 nm in accordance with the manufacturer's instructions. Protein concentrations were calculated from a standard curve with twofold serial dilutions with the highest standard of 500 pg/ml.

### 2.7 Immunocytochemistry

Mock‐infected or HSV‐1 infected TM cells were grown in 6‐well plates (without coverslips) until attainment of 70% to 80% confluency and subsequently subjected to TGF‐β1 (15 ng/ml) or Y‐27632 (10 μM) treatment for 12 h. To investigate the independent effects of candidate cytokines on cytoskeletal changes, serum‐starved TM cells were also treated with MCP‐1 (10 ng/ml or 50 ng/ml) or TGF‐β1 (15 ng/ml) for 15 min. After fixing, washing, permeabilization, nonspecific binding was blocked with 2.5% normal horse serum for 30 min. Pooled murine monoclonal anti‐RhoA antibody was applied (1:500 dilution) in 2.5% normal horse serum overnight at 4℃. Following thoroughly washing away the primary antibodies, secondary goat anti‐mouse antibody was subsequently applied for 1 h. Polymerized actin stress fibres were stained with rhodamine phalloidin in accordance with the manufacturer's instructions (Invitrogen). Cell nuclei were counterstained with 4′,6‐diamidino‐2‐phenylindole (DAPI). An inverted fluorescence microscope (IX83; Olympus) was used for imaging.

### 2.8 Statistical analyses

All data are expressed as means ±SD for at least three independent experiments unless otherwise mentioned. The significance of differences between groups was evaluated using Student's *t* test. One‐way analysis of variance was used for comparisons among three groups. *p* < 0.05 was taken to indicate a significant difference.

## 3 RESULTS

### 3.1 Inflammatory cytokine array

**FIGURE 1:**
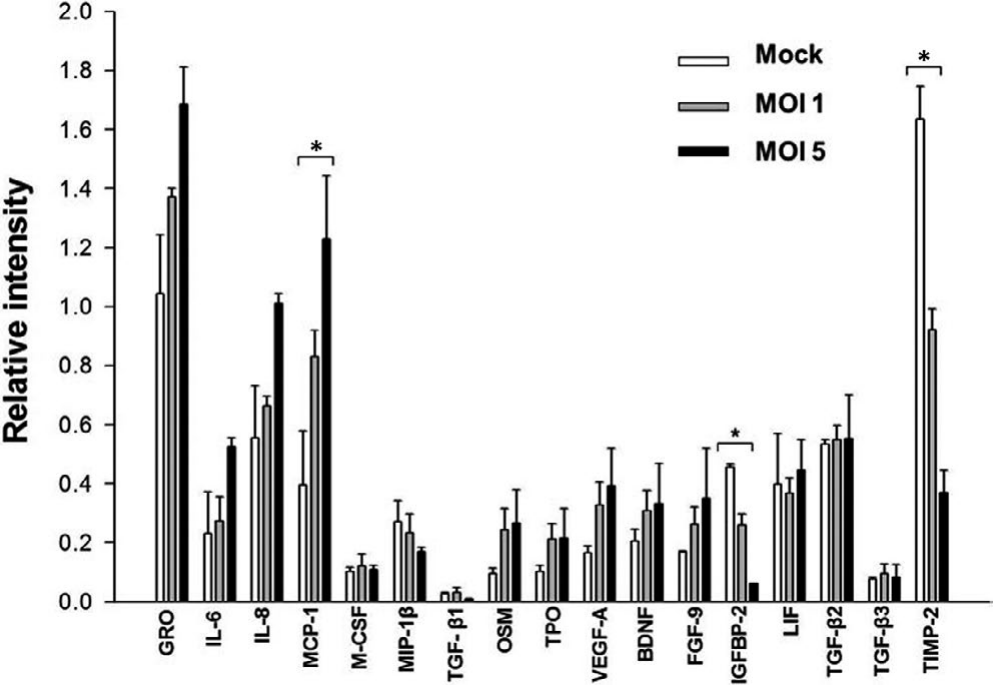
Profiles of inflammatory cytokines and chemokines induction by HSV‐1‐infection in human TM cells. Primary human TM cells were mock‐infected or infected with HSV‐1 at a MOI 1 or 5. At 12 h post‐infection, the supernatant of HSV‐1‐infected TM cells was assayed with a cytokine array. HSV‐1‐infected TM cells showed a significant stepwise increase in the expression of MCP‐1 and decrease in the expression of insulin‐like growth factor‐binding protein (IGFBP)‐2 and tissue inhibitor of metalloproteinase (TIMP)‐2, relative to mock‐infected TM cells. Data are shown as mean ±SD of triplicates. *p < 0.05 calculated using the independent *t* test. Abbreviation: GRO, growth‐regulated oncogene; IL, interleukin; MCP, monocyte chemoattractant protein; M‐CSF, macrophage colony‐stimulating factor; MIP, macrophage inflammatory protein; TGF, transforming growth factor; OSM, oncostatin M; TPO, thrombopoietin; VEGF, vascular endothelial growth factor; BDNF, brain‐derived neurotrophic factor, FGF, fibroblast growth factor; IGFBP, insulin‐like growth factor‐binding protein; LIF, Leukaemia inhibitory factor; and TIMP, tissue inhibitor of metalloproteinases

We initially used cytokine array analyses to gain a comprehensive understanding of the overall changes in inflammatory cytokines induced by HSV‐1 infection. For this purpose, primary human TM cells were mock‐infected or infected with HSV‐1 at a MOI 1 or 5. At 12 h PI, the supernatant of HSV‐1‐infected TM cells was assayed with a cytokine array. As shown in Figure 1, a significant stepwise increase in the expression of MCP‐1 was observed in HSV‐1 infected TM cells, compared with mock‐infected cells (*p* < 0.05). HSV‐1‐infected TM cells also showed significant stepwise decreases in the expression of insulin‐like growth factor‐binding protein (IGFBP)‐2 and tissue inhibitor of metalloproteinase (TIMP)‐2, relative to mock‐infected TM cells (all, *p* < 0.05).

### 3.2 Analyses of Rho‐signalling activity upon HSV‐1 infection

**FIGURE 2:**
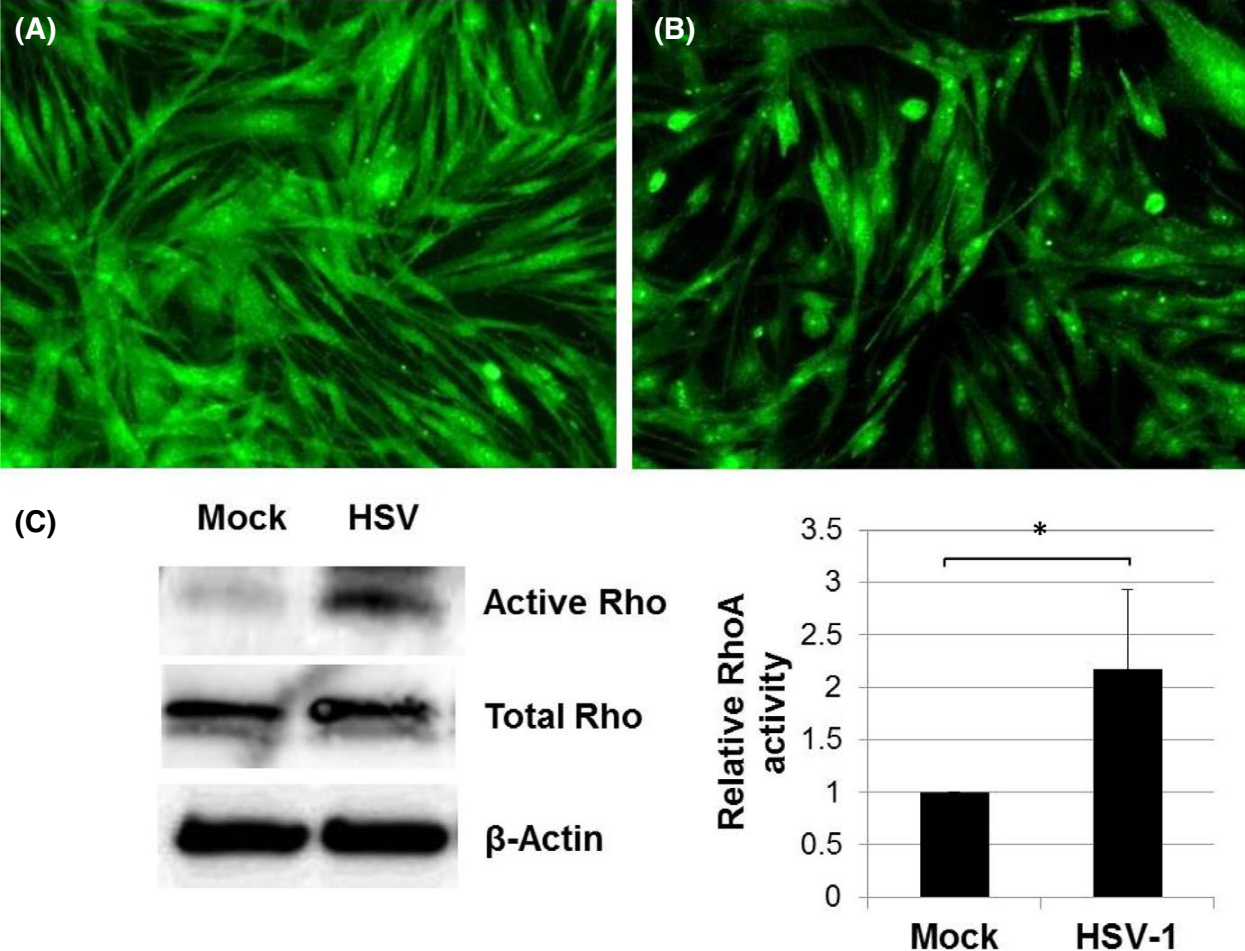
HSV‐1 infection induced activation of RhoA signalling pathways in primary human TM cells. Primary human TM cells were mock‐infected or infected with HSV‐1 at a MOI 1. At 12 h post‐infection, mock‐infected (A) and HSV‐1‐infected TM cells (B) were immunolabeled with anti‐RhoA antibody (green) and observed by fluorescence microscopy. RhoA was distributed throughout the cytoplasm, and HSV‐1 infection caused narrowing of the cytoplasm of TM cell processes. The RhoA activity assay shows twofold increase in HSV‐1 infected TM cells, compared with mock‐infected TM cells (C). The grouping of gels/blots cropped from the same gel. Data are shown as means ±SD, *n* = 4. *p < 0.05 calculated using the independent *t* test

To gain insight into the detailed mechanistic basis during HSV‐1 infection, GTP‐bound RhoA was measured using a pull‐down assay, and GTPase protein localization was visualized by immunofluorescence microscopy. We found that RhoA was distributed throughout the cytoplasm (Figure 2A), and HSV‐1 infection caused narrowing of the cytoplasm of TM cell processes (Figure 2B). When the RhoA activity assay was performed, a twofold increase in RhoA activity by HSV‐1 infection was observed (Figure 2C).

### 3.3 Synthesis of viral DNA upon HSV‐1 infection and treatment

We subsequently determined the effects of various agents on HSV‐1 replication. To address this aspect, human TM cells were infected at a MOI of 1 in the presence or absence of TGF‐β1, Y‐27632, or DEX treatment and total viral DNA was quantified at 12 h and 2 days PI.

**FIGURE 3:**
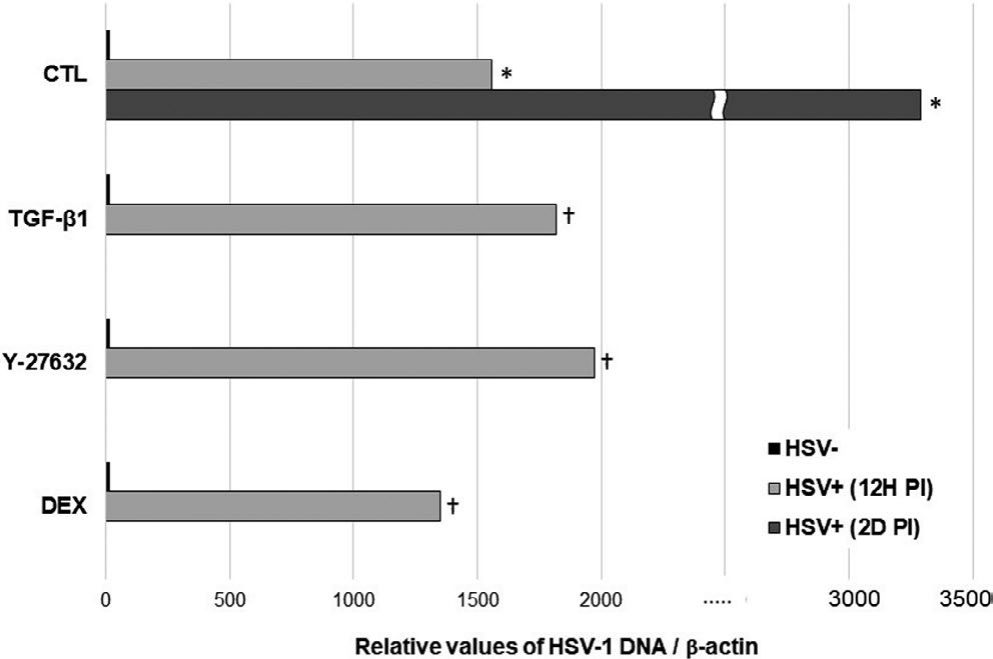
Viral DNA accumulation upon HSV‐1 infection in the presence or absence of TGF‐β1, Y‐27632 and dexamethasone (DEX) treatment. (^*^ *p* < 0.05 vs. transcripts from unstimulated mock‐infected cells; ^†^ *p* < 0.05 vs. transcripts from stimulated mock‐infected cells). The level of HSV‐1 DNA polymerase mRNA increased exponentially upon HSV‐1 infection at 12 h and 2 days PI. However, there were no significant differences in viral transcripts across the treatment groups at 12 h PI (*p* = 0.577, ANOVA). *N* = 3

As shown in Figure 3, the production of viral DNA increased exponentially upon HSV‐1 infection at 12 h and 2 days PI. However, there were no significant differences in viral transcripts across the treatment groups at 12 h PI (*p* = 0.577, ANOVA; Figure 3).

### 3.4 Expression of molecules induced by HSV‐1 infection and TGF‐β1, Y‐27632 and DEX treatment

**FIGURE 4:**
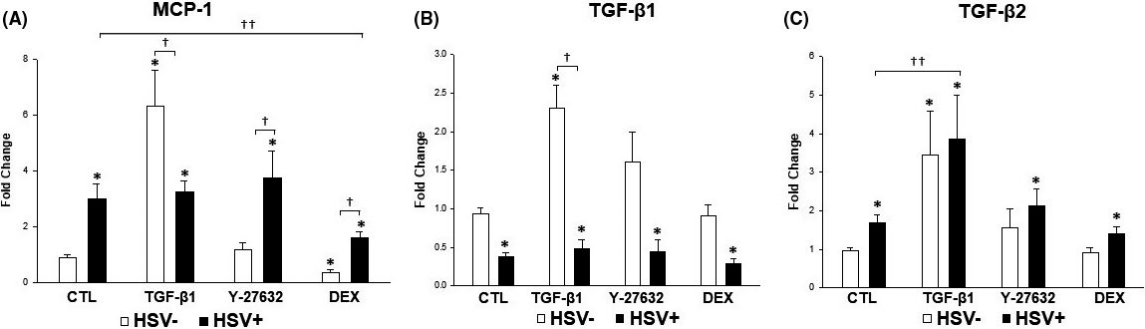
Quantitative determination of mRNA expression levels of MCP‐1, TGF‐β1 and TGF‐β2 according to HSV‐1 infection and treatment with TGF‐β1, Y‐27632 and dexamethasone (DEX). Cells were infected with HSV‐1 at a MOI of 1 in the presence or absence of various treatment and analysed at 12 h post‐infection. HSV‐1 infection significantly increased the expression of MCP‐1 (A), which was decreased by treatment with DEX. HSV‐1 infection also significantly increased the expression of TGF‐β2 (C), which was further increased by treatment with TGF‐β1. However, HSV‐1‐infected cells showed significantly decreased induction of mRNA transcripts encoding TGF‐β1 (B). Results are expressed as the mean ±standard deviation of three different experiments. (^*^ *p* < 0.05 vs. transcripts from unstimulated mock‐infected cells; ^†^ *p* < 0.05 vs. transcripts from stimulated mock‐infected cells; ^††^ *p* < 0.05 vs. transcripts from HSV‐1‐infected and unstimulated TM cells)

**FIGURE 5:**
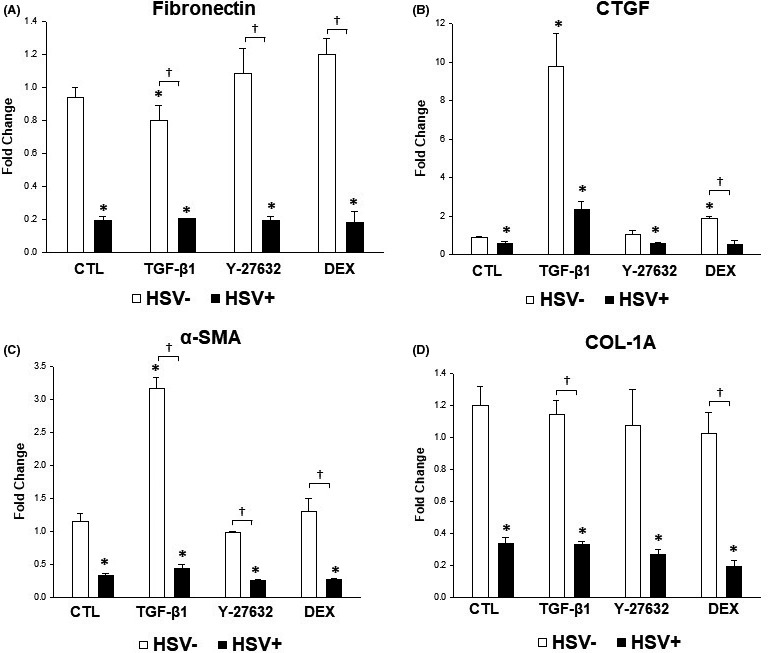
Quantitative determination of fibrogenic mRNA expression levels according to HSV‐1 infection and various treatment with TGF‐β1, Y‐27632 and dexamethasone (DEX). Cells were infected with HSV‐1 at a MOI of 1 in the presence or absence of various treatment and analysed at 12 h post‐infection. HSV‐1 infected cells showed decreased induction of many mRNA transcripts encoding fibrogenic matrix proteins. Expression of fibronectin (A), connective tissue growth factor (B), α‐smooth muscle actin (C) and collagen‐1A (D) were significantly decreased after HSV‐1 infection. Data are shown as mean ±SD of triplicates. (^*^ *p* < 0.05 vs. transcripts from unstimulated mock‐infected cells; ^†^ *p* < 0.05 vs. transcripts from HSV‐1‐infected and unstimulated TM cells)

To understand gene expression profiles upon HSV‐1 infection in the presence or absence of various treatments, real‐time PCR was performed to detect transcripts associated with elevation of IOP and inflammation (MCP‐1, TGF‐β1, TGF‐β2, fibronectin, α‐smooth muscle actin [SMA], collagen‐1A and connective tissue growth factor [CTGF]). Transcripts from cells under various experimental conditions were compared to those produced at baseline by uninfected and unstimulated TM cells. As expected, HSV‐1 infection significantly increased the expression of MCP‐1, which was decreased by treatment with DEX (*p* = 0.037; Figure 4A). HSV‐1 infection also significantly increased the expression of TGF‐β2, which was further increased by treatment with TGF‐β1 (*p* = 0.037; Figure 4C). On the other hand, HSV‐1 infected cells showed decreased induction of many mRNA transcripts encoding fibrogenic matrix proteins (fibronectin, α‐SMA, collagen‐1A, CTGF and TGF‐β1; Figure 4B and Figure 5A‐D). Treatment of mock‐infected TM cells stimulated with TGF‐β1 caused significant elevation of MCP‐1, TGF‐β1, TGF‐β2, α‐SMA and CTGF (Figures 4 and 5).

### 3.5 Enhanced release of MCP‐1 in HSV‐1 infected TM cells

**FIGURE 6:**
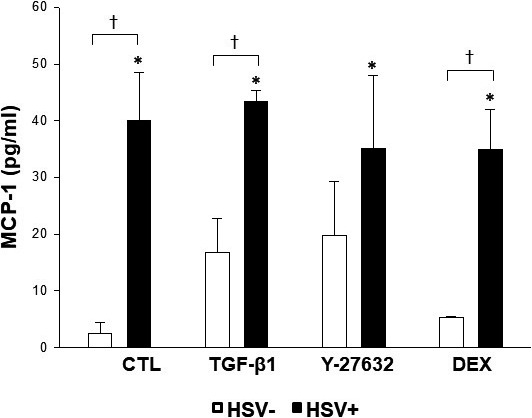
Enhanced secretion of MCP‐1 upon HSV‐1 infection in the presence or absence of TGF‐β1, Y‐27632 and dexamethasone (DEX) treatment in human TM cells. Cells were infected with HSV‐1 at a MOI of 1 in the presence or absence of various treatment and analysed at 12 h post‐infection. Supernatants were collected and the level of MCP‐1 was determined by ELISA. MCP‐1 expression showed a 40‐fold increase upon infection with HSV‐1, compared to unstimulated mock‐infected cells (^*^ *p* < 0.05 vs. MCP‐1 expression of unstimulated mock‐infected cells; ^†^ *p* < 0.05 vs. MCP‐1 expression of stimulated mock‐infected cells). Data are shown as mean ±SD of triplicates

Having found that the HSV‐1 infected TM cells exhibit increased expression of MCP‐1 by real‐time PCR and cytokine array, we validated the expression of MCP‐1 upon HSV‐1 infection and various treatments using ELISA assay. Consistent with the results of PCR analyses, MCP‐1 expression showed a 40‐fold increase upon infection with HSV‐1, compared to unstimulated mock‐infected cells (Figure 6).

### 3.6 Cytoskeletal integrity of TM cells induced by HSV‐1 infection and various treatments

**FIGURE 7:**
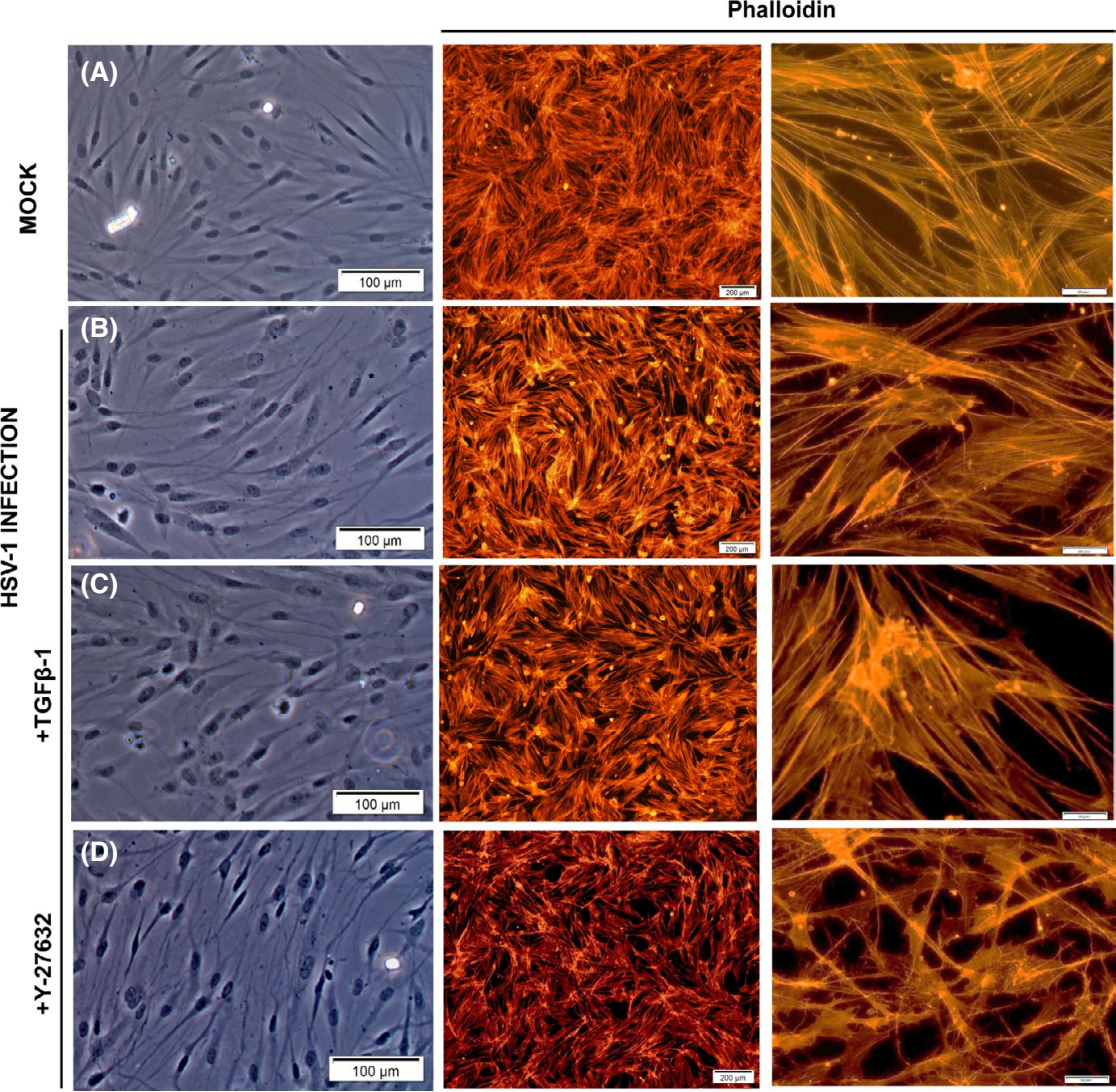
Effects of HSV‐1 infection on the cytoskeletal integrity of human TM cells in the presence or absence of TGF‐β1 or Y‐27632. Mock‐infected or HSV‐1 infected TM cells were grown in 6‐well plates until attainment of 70% to 80% confluency and subsequently subjected to TGF‐β1 (15 ng/ml) or Y‐27632 (10 μM) treatment for 12 h. The representative images of stained cells revealed an increase in actin stress fibres (red) after HSV‐1 infection (B), compared to mock infection (A). Moreover, the effects of HSV‐1 on actin stress fibres were further enhanced by the treatment with TGF‐β1 (C) and inhibited by treatment with Y‐27632 (D). Stress fibres were stained with rhodamine‐phalloidin (red signals). Representative bright‐field and immunostaining images of three independent analyses were shown

**FIGURE 8:**
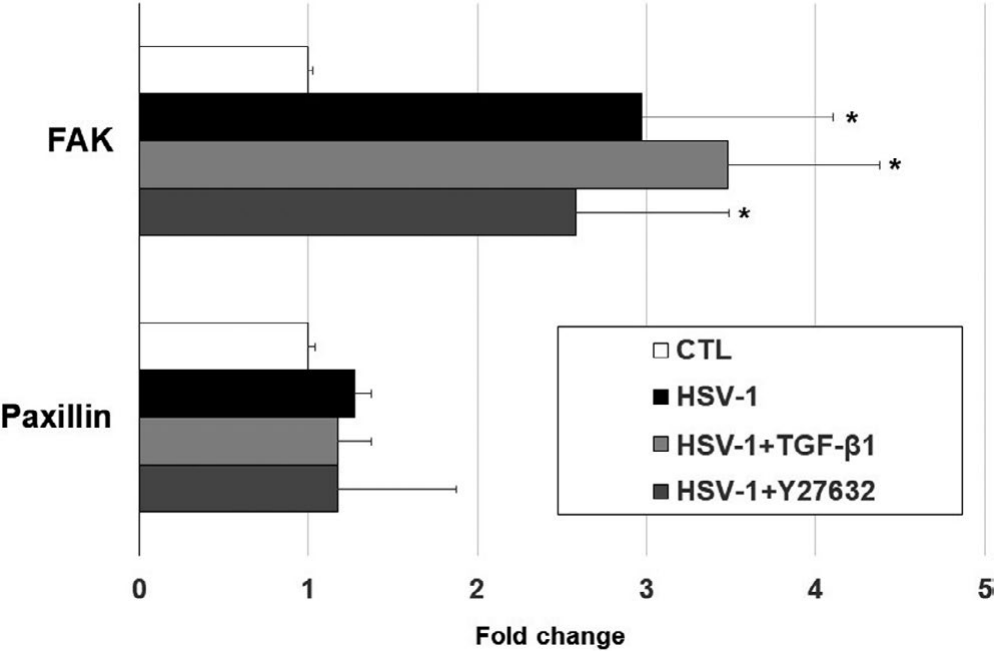
Quantitative determination of cell adhesion‐related mRNA expression levels in TM cells untreated or infected with HSV‐1 at MOI of 1 and/or stimulated with TGF‐β1 or Y‐27632. Results were normalized to β‐actin and quantitated as fold‐change compared to mRNA levels in uninfected, unstimulated TM cells. HSV‐1 infected cells (black bars) induced significant increase in focal adhesion kinase (FAK). Infected cells stimulated with TGF‐β1 (light grey bars) further increased the expression, whereas those stimulated with Y‐27632 (dark grey bars) decreased the expression of FAK. Data are shown as mean ±SD of triplicates

To understand the cytoskeletal changes associated with HSV‐1 infection in the presence or absence of various treatment, we examined the cellular morphology and the intracellular distribution of F‐actin in TM cells using fluorescent phalloidin. The representative images of stained cells revealed an increase in actin stress fibres (red) after HSV‐1 infection (Figure 7B), compared to mock infection (Figure 7A). Moreover, the effects of HSV‐1 infection on F‐actin formation were enhanced by treatment with TGF‐β1 (Figure 7C) and inhibited by treatment with Y‐27632 (Figure 7D). Real‐time PCR analyses regarding the cell adhesion molecules showed that focal adhesion kinase (FAK) was significantly increased after HSV‐1 infection. Infected cells stimulated with TGF‐β1 showed further increased expression of FAK, whereas infected cells stimulated with Y‐27632 decreased the expression of FAK (Figure 8).

### 3.7 Effects of MCP‐1 on cytoskeletal integrity of TM cells

**FIGURE 9:**
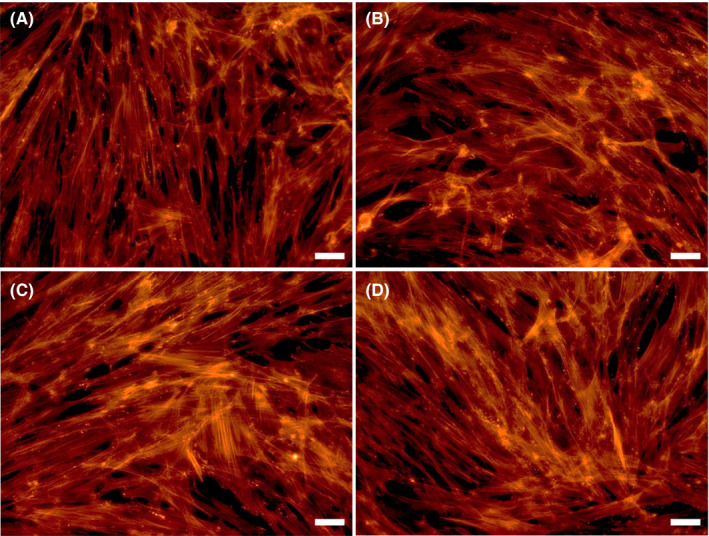
Effects of MCP‐1 and TGF‐β1 treatment on the cytoskeletal integrity of human TM cells. TM cells were grown in 6‐well plates until attainment of 70% to 80% confluency, and serum‐starved human TM cells were subsequently subjected to different concentrations of MCP‐1 (10 ng/ml or 50 mg/ml) or TGF‐β1 (15 ng/ml) treatment. Normal serum‐starved TM cells (A), TM cells treated with 10 ng/ml MCP‐1 (B), TM cells treated with 50 ng/ml MCP‐1 (C) and TM cells treated with 15 ng/ml TGF‐β1 (D). The representative images of the stained cells revealed a dose‐dependent increase in actin stress fibres with MCP‐1 treatment, compared with the untreated TM cells (A–C), which was comparable to TM cells treated with 15 ng/ of TGF‐β1 (D). F‐actin stress fibres were stained with rhodamine‐phalloidin (red signals). Representative immunostaining images of three independent analyses were shown. *Scale bar*. 20 μm

Having found that HSV‐1 enhances cellular contractile activity in concert with increased release of MCP‐1 in human TM cells, we investigated the independent effects of target cytokines on cellular contractile activity. To this end, serum‐starved human TM cells were treated with different concentrations of MCP‐1 (10 ng/ml or 50 mg/ml) or TGF‐β1 (15 ng/ml). Representative images of stained cells revealed a dose‐dependent increase in actin stress fibres (*red*) with MCP‐1 treatment, compared with the untreated TM cells (Figure 9A–C), which was comparable to TM cells treated with 15 ng/ of TGF‐β1 (Figure 9C‐D).

## 4 DISCUSSION

The broad objective of this study was to gain our molecular understanding of a potential mechanism underlying IOP elevation in HSV‐1 viral anterior uveitis. IOP is finely regulated by the two‐way interplay between TM cell contractile activity and components of the ECM.^18^ In general, TM cells are contractile, and actin fibres are directly involved in normal and pathological conditions related to glaucoma.^10^ Glaucomatous eyes exhibit upregulation of genes related to cellular contractile activity and matrix function.^19^ Our data demonstrate that HSV‐1 infection induced cytoskeletal contraction in human TM cells, inducing increased expression of actin stress fibres (Figure 7), RhoA activity (Figure 2) and cell adhesion molecules (Figure 8). HSV‐1 activates the Rho‐GTPase signalling pathway within a target cell that helps facilitate viral entry and exploits actin and actin‐associated myosin components for viral entry.^20^, ^21^ Interestingly, fibronectin and fibrogenic genes such as α‐SMA, CTGF and TGF‐β1 were significantly downregulated by HSV‐1 infection in TM cells (Figures 4, 5). Our results suggest that HSV‐1 infection in TM cells increases cell contractile activity rather than fibrogenic activity. The increased TM cell contractile activity upon HSV‐1 infection may be responsible for the clinical manifestations of HSV‐1‐associated anterior uveitis, showing acute IOP elevation in the presence or inflammation, which resolves after cessation of inflammation.

When we investigated the overall changes in inflammatory cytokine profiles upon HSV‐1 infection, MCP‐1 was particularly upregulated in a dose‐dependent manner in HSV‐1 infected TM cells (Figure 1). The real‐time PCR and ELISA results validated the significant increase in MCP‐1 expression upon HSV‐1 infection in TM cells (Figures 4A and 6). Our results are in line with a previous study of Tumpey et al.,^22^ who found that MCP‐1 was upregulated during HSV‐1 ocular infection. MCP‐1 plays an important role in the pathogenesis of herpes stromal keratitis, and MCP‐1 secreted from HSV‐1‐infected keratocytes attracts CD4^+^ T cells into the cornea.^23^ Ohira et al.^24^ also reported that eyes with uveitic glaucoma show significantly elevated MCP‐1 levels in the aqueous humour compared to eyes with primary open‐angle glaucoma.

The chemokine MCP‐1 is a key mediator of inflammatory processes.^25^ It is secreted by fibroblasts, endothelial cells, vascular smooth muscle cells, monocytes, T cells and other cell types that mediate the influx of monocytes and macrophages to sites of inflammation.^26^ It is also involved in cytoskeletal remodelling in vascular smooth muscle cells with regard to mediating cell migration.^27^ In the present study, MCP‐1 induced the actin cytoskeleton in a dose‐dependent manner, which was comparable to TGF‐β1 (Figure 9). Notably, TM cells are mesenchymal cells originating from the neural crest^28^ and show characteristics of four different cell types (ie vascular endothelial cells, macrophages, fibroblasts and smooth muscle cells) in the fine regulation of IOP.^9^ Thus, the increased MCP‐1 level during HSV‐1 infection may increase contractile activity in human TM cells. Normal human TM cells secrete significant amounts of MCP‐1 even in the absence of any stimulation.^29^ On the other hand, MCP‐1 also increases the permeability of Schlemm's canal endothelial cells and has been shown to disrupt gap junctions between Schlemm's canal endothelial cells.^30^ Hence, further studies are required to comprehensively characterize the effects of MCP‐1 on the outflow system in hypertensive viral anterior uveitis.

TGF‐β plays a cardinal role in the pathogenesis of primary open‐angle glaucoma. The level of TGF‐β is highly elevated in the aqueous humour of glaucoma patients compared to healthy controls.^31^ As expected, treatment with TGF‐β1 increased the expression of various fibrogenic molecules, such as CTGF and α‐SMA (Figure 5B,C). TGF‐β1 also enhanced the expression of TGF‐β2 in HSV‐1 infected TM cells (Figure 4C). Therefore, a high concentration of TGF‐β in the human aqueous humour may further augment the effects of HSV‐1 infection in TM cells.

Patients with hypertensive anterior uveitis exhibit a good clinical response to treatment with topical steroids, which are the most commonly used agents for this disorder. We showed that DEX decreased the expression of MCP‐1 that was enhanced by HSV‐1 infection in TM cells (Figure 4A). In a previous study, we showed that DEX decreased the TGF‐β1 level that was enhanced by CMV infection in human TM cells.^32^ Therefore, the prompt response to DEX in hypertensive anterior uveitis seems to be mediated by suppression of the cytokine responsible for acute IOP elevation. However, frequent use of steroids can potentially cause steroid‐induced glaucoma.^33^ Consistent with this possibility, we showed that mock‐infected TM cells with DEX treatment significantly increased the expression of CTGF (Figure 5B), the protein which induces ECM synthesis and contractile activity in human TM cells.

Interestingly, we found that the increased contraction of TM cells by HSV‐1 infection involved activation of RhoA (Figure 2C), and the virus‐induced contraction were significantly ameliorated by addition of the ROCK inhibitor Y‐27632 (Figure 7D). Rho‐A signalling mediates TGF‐β‐induced IOP elevation in the early phase, but does not affect the sustained IOP elevation caused by TM fibrosis.^12^ As HSV‐1 infection causes generalized suppression of fibrogenic molecules (Figure 5), the acute IOP elevation induced by HSV‐1 infection seems to be mediated by increased contractile activity of TM cell, rather than ECM modulation. Kusuhara et al.^34^ who studied the efficacy of ROCK inhibitor treatment in uveitic glaucoma reported that some patients responded to the treatment, while others did not. It is assumed that there are subtypes of uveitic glaucoma in which TM cell contractions are the dominant mechanism of IOP elevation. Thus, ROCK inhibitor treatment may be effective for the management of patients with hypertensive anterior uveitis associated with increased TM cell contractile activity.

Regarding the cell contractile activity and stiffness, it is known that a certain percentage of TM cells can undergo cytoskeletal rearrangements from linear stress fibres to form distinct dome‐like structure known as cross‐linked actin networks (CLAN).^35^ This structure occurs in only 4% of confluent normal TM cells, whereas approximately 25% of confluent cultured primary human glaucomatous TM cells form CLAN. In this study, we did not observe definite manifestation of the CLAN formation in the HSV‐1 infected TM cells. CLAN has been reported to be induced in a dose‐dependent manner by prolonged treatment with dexamethasone or TGF‐β2.^35^ The formation of CLAN has been reported to be induced by 7 days of treatment with dexamethasone^36^ or 10 days of treatment with TGF‐β2.^37^ Direct cytopathic effects of HSV‐1 hinder long‐term observation of virus‐infected cells *in vitro*. Therefore, it would be necessary to conduct *in vivo* study to understand long‐term effects associated with viral infection in TM cells.

In conclusion, HSV‐1 infection induced increased cell contractile activity in human TM cells. ROCK inhibitor reverses the increased contraction. Enhanced TM cell contractile activity in concert with increased release of MCP‐1 in human TM cells is thought to responsible for the clinical manifestations of HSV‐1 anterior uveitis, showing marked IOP elevation at the time of inflammation.

## CONFLICT OF INTEREST

The authors confirm that there are no conflicts of interest.

## AUTHOR CONTRIBUTIONS

**Jin A Choi:** Conceptualization (lead); Funding acquisition (lead); Investigation (equal); Methodology (supporting); Resources (lead); Writing‐original draft (lead). **Hyun‐hee Ju:** Data curation (lead); Formal analysis (supporting); Methodology (lead); Project administration (supporting); Software (lead); Visualization (lead). **Ju‐Eun Kim:** Data curation (supporting); Formal analysis (supporting); Methodology (equal); Resources (supporting); Visualization (supporting); Writing‐original draft (supporting). **Jiyoung Lee:** Investigation (supporting). **Donghyun Jee:** Investigation (supporting). **Chan Kee Park:** Conceptualization (lead); Resources (supporting); Supervision (supporting); Writing‐review & editing (supporting). **Soon‐young Paik:** Conceptualization (lead); Funding acquisition (lead); Resources (lead); Writing‐review & editing (supporting).

## Supporting information

Supplementary Material

Supplementary Material

## Data Availability

Data available in article supplementary material.
