# Supplementary material for: Cytokine profile and cytoskeletal changes after herpes simplex virus type 1 infection in human trabecular meshwork cells
Source: J Cell Mol Med. 2021 Sep 1;25(19):9295–305. doi: 10.1111/jcmm.16862 (PMC8500954; doi:10.1111/jcmm.16862)
Supplement: Supplementary file 1 — Supplementary Material [file JCMM-25-9295-s001.pdf]

Supplementary information 1. The percent identities of major genes of HSV-1 between the clinical strain HSV-1 NCCP no. 43002 in comparison with HSV-1 KOS strain

Herpes Simplex Virus 1 glycoprotein					Per. Ident
Gene	Protein	cds (bp)	functions		
UL1	Glycoprotein L	675	Surface and membrane	Human herpesvirus 1 isolate KOS, complete genome	99.85%
UL22	Glycoprotein H	2517	Surface and membrane	Human herpesvirus 1 isolate KOS, complete genome	99.84%
RS1	Immediate early genes	3896	Major transcriptional activator. Essential for progression beyond the immediate-early phase of infection	Human herpesvirus 1 isolate KOS, complete genome	99.95%
UL42	DNA polymerase	1466	DNA polymerase processivity factor	Human herpesvirus 1 isolate KOS, complete genome	99.79%

The primer sequence of each gene

Region	No.	Primer	Primer sequence	length	TM	position	size	sequence
UL1	1	UL1_F	ATG GGG ATT TTG GGT TGG GTC G	22	64.0	1~22	675	ATGGGGATTTTGGGTTGGGTCGGGCTTATTGCCGTTGGGGTTTTGTGTGTGCGGGGGGGCTTGTCTTCAACCGAATATGT<br>TATTCGGAGTCGGGTGGCTCGAGAGGTGGGGGATATATTAAAGGTGCCTTGTGTGCCGCTCCCGTCTGACGATCTTGATT<br>GGCGTTACGAGACCCCCTCGGCTATAAACTATGCTTTGATAGACGGTATATTTTTGCGTTATCACTGTCCCGGATTGGAC
		UL1_R	TTA GAG GCG CCG GGA GTG G	19	63.8	657~675		
UL22	1	UL22_F1	TTA TTC GCG TCT CCA AAA AAA CGG [illegible]	25	65.8	1~25	844	[illegible]GCGGCGGTAATCATAACCA<br>CGCCCAGCGCAGAGGCGGCCAGAAACCCGGGCGCAATTGCGGCCACGGGCTGCGTGTCAAAGGCTAGCAAATGAATGACG<br>GTTCCGTTTGGAAATAGCAACAAGGCCGTGGACGGCACGTCGCTCGAAAACACGCTTGGGGCGCCCTCCGTCGGCCCGGC<br>GGCGATTTGCAGCTGTGTGTTGTCCGTATCCACCAGCAACACAGACATGACCTCCCCGGCCGGGGTGTAGCGCATAAACA<br>CGGCCCCCACGAGCCCCAGGTCGCGCTGGTTTTGGGTGCGCACCAGCCGCTTGGACTCGATATCCCGGGTGGAGCCTTCG<br>CATGTCGCGGTGAGGTAGGTTAGGAACAGTGGGCGTCGGACGTCGACGCCGGTGAGCTTGTAGCCGATCCCCCGGGGCAG<br>AGGGGAGTGGGTGACGACGTAGCTGGCGTTGTGGGTGATGGGTACCAGGATCCGTGGCTCGACGTTGGCAGACTGCCCCC<br>CGCACCGATGTGAGGCCTCAGGGACGAAGGCGCGGATCAGGGCGTTGTAGTGTGCCCAGCGCGTCAGGGTCGAGGCGAGG<br>CCGTGGGTCTGCTGGGCCAGGACTTCGACCGGGGTCTCGGATCGGGTGGCTTGAGCCAGCGCGTCCAGGATAAACACGCT<br>CTCGTCTAGATCAAAGCGCAGGGAGGCCGCGCATGGCGAAAAGTGGTCCGGAAGCCAAAAGAGGGTTTTCTGGTGGTCGG<br>CCCGGGCCAGCGCGGTCCGGAGGTCGGCGTTGGTCGCTGCGGCGACGTCGGACGTACACAGGGCCGAGGCTATCAGAAGG
		UL22_R1	ACC TAC CTC ACC GCG ACA TGC	22	64.0	823~844		
	2	UL22_F2	CCA GCA ACA CAG ACA TGA CCT C	23	62.9	693~715	827	
		UL22_R2	CGC GAA CAG AGC TTG GCA TTA CA	22	64.0	1498~1519		
	3	UL22_F3	CCC CTA GAA ACT CGG CAT ACA G	23	64.0	1387~1409	859	
		UL22_R3	AGG TAG GTA CGT GTA TTT CTC CC	22	64.0	2224~2245		
	4	UL22_F4	GGC TGT CGT GCA TGG ATA TCA C	21	65.3	2098~2118	420	
		UL22_R4	ATG GGG AAT GGT TTA TGG TTC GTG G	25	64.2	2493~2517		
RS1	1	RS1_F1	ATG GCG TCG GAG AAC AAG CAG	21	63.3	1~21	932	[illegible]CCACAACG<br>TCCCGTCGCGGGGGCGTGGCCAAGCCCGCCTCCGCCCCCAGCACCTCCACGGCCCCCGCCGCCGCCAGCA<br>CGGTGCCGCTGCGGCCCGTGGCCGAGGCCCAGCGAATCCCGGGCGGCGCCGGCGGCAGGGCCCCCGGGCC<br>GTCGTCGTCGTCGCCGCGCAGCACCAGCGGGGGGGCGTCGTCGTCGGGCTCCAGCAGGGCGCGGGCGCAA<br>AAGTCCCTCCGCGGCCCGCGCCACCGGGCCGGGCCGGCGCGCACCGCCTCGCGCCCCAGCGCCACGTACA<br>CGGGCCGCAGCGGCGCGCCCAGGCCCCAGCGCGCGCAGGCGCGGTGCGAGTGGGCCTCCTCCTCGCAGAA<br>GTCCGGCGCGCCGGGCGCCATGGCGTCGGTGGTCCCCGAGGCCGCCGCCCGGCCGTCCAGCGCCGGCAGC<br>ACGGCCCGGCGGTACTCGCGCGGGGACATGGGCACCGGCGTGTCCGGGCCGAAGCGCGTGCGCACGCGGT<br>AGCGCACGTTGCCGCCGCGGCACAGGCGCAGCGGCGGCGCGTCGGGGTACAGGCGCGCGTGCGCGGCCTC<br>CACGCGCGCGAAGACCCCCGGGCCGAACACGCGGCCCGAGGCCAGCACCGTGCGGCGCAGGTCCCGCGCC<br>GCCGGCCAGCGCACGGCGCACTGCACGGCGGGCAGCAGGTCGCACGCCAGGTAGGCGTGCTGCCGCGACA<br>CCGCGGGCCCGTCGGCGGGCCAGTCGCAGGCGCGCACGGTGTTGACCACGATGAGCCGCCGGTCGCCGGC<br>GCTGGCGAGCAGCCCCAGAAACTCCACGGCCCCGGCGAAGGCCAGGTCCCGCGTGGACAGCAGCAGCACG
		RS1_R1	ACG TAC CCG TCG CGA TAG C	19	61.7	914~932		
	2	RS1_F2	TCG AGC TGG ACG CCG ACG	18	63.0	872~889	860	
		RS1_R2	CTC CAG GAT CCC GCG GCA	18	63.0	1714~1731		
	3	RS1_F3	CGC CGG CGC AGA TGA CGA	18	63.0	1479~1498	720	
		RS1_R3	CCG GGA CTC TTG CGC TTG C	19	63.8	2180~2198		
	4	RS1_F4	CGG ACC TGC TGT TTG ACA ACC A	22	64.0	2063~2084	859	
		RS1_R4	GCA CAG CCG GTT GGC CAG	18	63.0	2902~2921		
	5	RS1_F5	GCG TGG TGG TGC TGT ACT CG	20	64.6	2786~2805	1112	
		RS1_R5	TTA CAG CAC CCC GTC CCC	18	60.7	3880~3897		
UL42	1	UL42_F1	ATG ACG GAT TCC CCT GGC G	19	61.7	1~19	823	ATGACGGATTCCCCTGGCGGTGTGGCCCCCGCCTCCCCCGTGGAGGACGCGTCGGACGCGTCCCTCGGGCAGCCGGAGGA<br>GGGGGCGCCCTGCCAGGTGGTCCTGCAGGGCGCCGAACTTAATGGAATCCTACAGGCGTTTGCCCCGCTGCGCACGAGCC<br>TTCTGGACTCGCTTCTGGTTATGGGCGACCGGGGCATCCTTATCCATAACACGATCTTTGGGGAGCAGGTGTTCCTGCCC<br>CTGGAACACTCGCAATTCAGTCGGTATCGCTGGCGCGGACCCACGGCGGCGTTCCTGTCTCTCGTGGACCAGAAGCGCTC<br>CCTCCTGAGCGTGTTTCGCGCCAACCAGTACCCGGACCTACGTCGGGTGGAGTTGGCGATCACGGGCCAGGCCCCGTTTC
		UL42_R1	GC ATG CTG CAA TCG TCG ACG A	21	63.3	803~823		
	2	UL42_F2	GCA CCC AGG TCC AGA TCC T	19	61.7	698~716	770	
		UL42_R2	GG GAA TCC AAA ACC ATA CGG G	21	61.3	1443~1467		
