# Supplementary material for: Cytokine profile and cytoskeletal changes after herpes simplex virus type 1 infection in human trabecular meshwork cells
Source: J Cell Mol Med. 2021 Sep 1;25(19):9295–305. doi: 10.1111/jcmm.16862 (PMC8500954; doi:10.1111/jcmm.16862)
Supplement: Supplementary file 2 — Supplementary Material [file JCMM-25-9295-s002.docx]

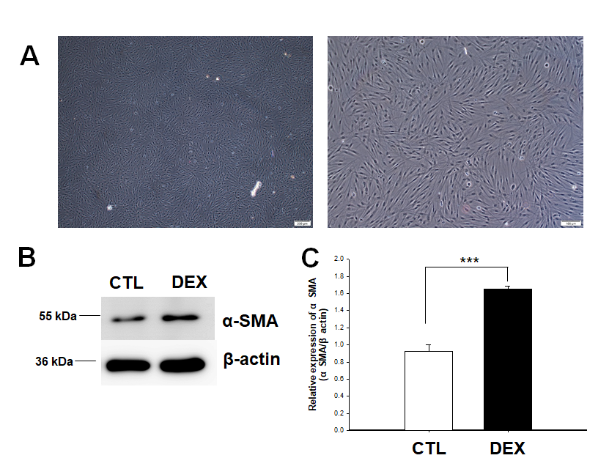


**Supplementary information 2.** **Characterization of TM cells.** The confluent TM cells show a cobblestone-like pattern with some overlapping processes in the low and high magnification microscopy (A). Next, the serum-starved TM cells subjected to the treatment with dexamethasone (500 nM/ 4 days). Immunoblot analyses confirmed that the untreated control TM cells exhibit the expression of α-smooth muscle action (SMA), which is significantly increased upon the treatment with dexamethasone (B). Histograms depict the fold change in the levels of α-SMA in the TM cells treated with dexamethasone, based on densitometric analysis. Values are mean ± SEM, n=3, ****P* ≤ 0.001.
